# Supplementary material for: Somatization symptomology and its association with stress in patients with irritable bowel syndrome
Source: PLoS One. 2025 Jan 6;20(1):e0312506. doi: 10.1371/journal.pone.0312506 (PMC11703010; doi:10.1371/journal.pone.0312506)
Supplement: S1 Table — This is the table that represents the different PHQ scores in each age range for the IBS sample. (DOCX) [file pone.0312506.s004.docx]

| SSRS score (Age) |  |  | Frequency  (N) | Percentage | Mean  (IBS) | Std. Dev  (IBS) | Std. Error |
| --- | --- | --- | --- | --- | --- | --- | --- |
|  |  | 18-29 | 19 | 28.4 | 72.58 | 17.65 | 4.049 |
|  |  | 30-39 | 15 | 22.4 | 75.27 | 19.16 | 4.948 |
|  |  | 40-49 | 18 | 26.9 | 78.44 | 16.36 | 3.85711 |
|  |  | 50-59 | 9 | 13.4 | 68.89 | 27.63804 | 9.21268 |
|  |  | 60-69 | 6 | 9.0 | 83.17 | 23.92837 | 9.76871 |
| Total |  |  | 67 | 100.0 | 75.20 | 19.59 | 2.39 |

**Table S1. Stress related somatization burden by age in IBS patients**
