# Supplementary material for: Somatization symptomology and its association with stress in patients with irritable bowel syndrome
Source: PLoS One. 2025 Jan 6;20(1):e0312506. doi: 10.1371/journal.pone.0312506 (PMC11703010; doi:10.1371/journal.pone.0312506)
Supplement: Attachment — Submitted filename: Recommendation.docx [file pone.0312506.s005.docx]

**Recommendations**

**Title and Abstract**

- **Title:**
  - "Somatization Symptomology and Its Association with Stress in Patients with Irritable Bowel Syndrome," is clear and accurately reflects the study's focus. No changes are necessary.
- **Abstract:**
  - The abstract provides a concise overview of the study. However, it lacks specific details about the sample characteristics and methods.
  - **Recommendations:**
    - Include the mean age and gender distribution of the participants.
    - Summarize the key statistical methods used, such as linear regression analysis.
    - Provide a brief mention of the main findings with specific statistical results (e.g., p-values, confidence intervals).

**Introduction**

- The introduction effectively establishes the importance of studying the overlap between IBS and SSD and the potential role of stress. However, it could benefit from a more detailed background.
- **Recommendations:**
  - Expand on the physiological and psychological mechanisms that may link stress and somatization in IBS patients. Include references to relevant theories and previous research.
  - Provide a more comprehensive review of existing literature, highlighting key studies that have investigated the relationship between stress, somatization, and IBS.

**Methods**

- **Sampling Method:**
  - The study mentions the use of purposeful sampling but does not provide detailed criteria for participant selection.
  - **Recommendations:**
    - Clearly describe the inclusion and exclusion criteria for selecting participants. This should include diagnostic criteria for IBS and SSD, as well as any other relevant factors.
    - Justify the sample size of 100 participants. A power analysis should be conducted to determine if the sample size is adequate to detect significant effects.
- **Data Collection Tools:**
  - The use of PHQ-15 and SSRS is appropriate, but their reliability and validity in this specific population are not discussed.
  - **Recommendations:**
    - Discuss the reliability (e.g., Cronbach's alpha) and validity (e.g., construct validity) of the PHQ-15 and SSRS within the context of this study population. Mention if these tools have been validated in previous research involving IBS or SSD patients.
- **Statistical Analysis:**
  - The paper mentions the use of SPSS for data analysis but lacks specifics on the statistical tests performed.
  - **Recommendations:**
    - Provide a detailed description of the statistical methods used. This should include descriptive statistics, inferential statistics (e.g., t-tests, ANOVA), and the specific type of linear regression analysis conducted.
    - Include information on how missing data were handled and any assumptions checked for the regression analysis.

**Results**

- **Demographic Information:**
  - The results section does not provide detailed demographic information, which is crucial for understanding the sample.
  - **Recommendations:**
    - Include a table summarizing the demographic characteristics of the sample, such as mean age, gender distribution, and other relevant variables (e.g., duration of illness, comorbid conditions).
- **Presentation of Results:**
  - The results for PHQ and SSRS scores are presented but lack comprehensive statistical details.
  - **Recommendations:**
    - Present the range, mean, and standard deviation of PHQ and SSRS scores for each group (IBS, SSD, IBS with SSD).
    - Include regression coefficients, confidence intervals, and p-values for the linear regression analysis. This will provide a clearer understanding of the relationship between stress and somatization symptoms.
- **Interpretation of Findings:**
  - The results indicate a stronger correlation between stress-related somatic symptoms and somatization in elderly patients but lack a detailed discussion on this point.
  - **Recommendations:**
    - Discuss possible reasons why stress-related somatic symptoms are more pronounced in elderly IBS patients. This could include factors such as age-related physiological changes, comorbidities, or social factors.
    - Consider exploring whether the severity of IBS symptoms moderates the relationship between stress and somatization.

**Conclusion**

- The conclusion summarizes the main findings and suggests targeted psycho-social interventions but lacks a discussion on limitations and future research directions.
- **Recommendations:**
  - Discuss the limitations of the study. These could include the sample size, the use of self-reported measures, and the generalizability of the findings.
  - Suggest directions for future research. This could involve longitudinal studies to assess causality, exploring interventions in a more diverse population, or examining the impact of different types of stress on somatization symptoms in IBS patients.

Comments:

**Title and Abstract:** The abstract lacks specific details about the sample characteristics and methods. For example, it does not provide the mean age and gender distribution of the participants, nor does it summarize the key statistical methods used. Including these details would make the abstract more informative and comprehensive.

**Introduction:** Although the introduction highlights the relevance of the study, it lacks a detailed background on the mechanisms linking stress and somatization in IBS patients. Additionally, the review of existing literature is not comprehensive. The authors should expand on the physiological and psychological mechanisms that may link stress and somatization in IBS patients and provide a more detailed review of relevant studies.

**Methods:** The methods section does not provide sufficient detail on the sampling method. The authors mention purposeful sampling but do not describe the inclusion and exclusion criteria for participant selection. Furthermore, the sample size of 100 participants is not justified, and there is no discussion on the reliability and validity of the data collection tools (PHQ-15 and SSRS). The authors should also provide more information on the statistical tests used, such as descriptive statistics and the specific type of linear regression analysis conducted.

**Results:** The results section is missing detailed demographic information, such as the mean age and gender distribution of the participants. Additionally, the range of scores and detailed statistical results (e.g., regression coefficients, confidence intervals, p-values) are not presented. Including these details would provide a clearer understanding of the data and the relationship between stress and somatization symptoms.

**Conclusion:** The conclusion summarizes the main findings but does not discuss the limitations of the study or suggest directions for future research. The authors should address the study's limitations, such as the sample size and potential biases, and suggest areas for future research, such as exploring interventions in a more diverse population or conducting longitudinal studies to assess causality.

**Summary:** In summary, the article has several shortcomings, including insufficient details in the abstract, an inadequate review of relevant literature in the introduction, lack of clarity in the methods section, incomplete presentation of results, and a conclusion that does not address limitations or future research directions. These issues need to be addressed to make the article more comprehensive, transparent, and informative.

Thank you for the opportunity to review your work.
