# Supplementary material for: Somatization symptomology and its association with stress in patients with irritable bowel syndrome
Source: PLoS One. 2025 Jan 6;20(1):e0312506. doi: 10.1371/journal.pone.0312506 (PMC11703010; doi:10.1371/journal.pone.0312506)
Supplement: Attachment — Submitted filename: Recommendations.docx [file pone.0312506.s006.docx]

**Recommendations**

• **Stratify Participant Groups**:

- Consider stratifying the sample into more specific subgroups (e.g., IBS only, SSD only, IBS with SSD) to reduce heterogeneity and allow for clearer insights into each group’s symptomatology and stress responses.

• **Detail Statistical Methods**:

- Provide a comprehensive description of all statistical methods used, including the rationale for their selection, to enhance transparency and reproducibility.

• **Enhance Results Presentation**:

- Report additional statistical details such as confidence intervals, p-values, regression coefficients, and R-squared values to provide a more complete understanding of the findings.

**Contextualize Findings with Literature**:

- Include a robust literature review to situate the study’s findings within the broader context of existing research, enhancing the discussion and highlighting the study’s contribution.
