# Supplementary material for: Somatization symptomology and its association with stress in patients with irritable bowel syndrome
Source: PLoS One. 2025 Jan 6;20(1):e0312506. doi: 10.1371/journal.pone.0312506 (PMC11703010; doi:10.1371/journal.pone.0312506)
Supplement: Attachment — Submitted filename: Response to reviewers.docx [file pone.0312506.s007.docx]

**Reviewer 1**

**Recommendations**

**Title and Abstract**

- **Title:**
  - "Somatization Symptomology and Its Association with Stress in Patients with Irritable Bowel Syndrome," is clear and accurately reflects the study's focus. No changes are necessary.
- **Abstract:**
  - The abstract provides a concise overview of the study. However, it lacks specific details about the sample characteristics and methods.
  - **Recommendations:**
    - Include the mean age and gender distribution of the participants.

Response: Abstract has been updated. We have included frequencies and percentages of participants now as initially how the data was entered in the SPSS spreadsheet; it was coded rather than entering original ages of each participant. Participants in the age range 18-29 years is coded as 1, 30-39 years as category 2, 40-49 years as category 3, 50-59 as category 4, 60-69 as category 5. Similarly, gender distribution has been included now. There were more males than females in all three samples.

- Summarize the key statistical methods used, such as linear regression analysis.

Response: Linear regression used was a simple linear regression. In other words, it was linear bivariate regression. It has been included in the manuscript now especially under sample size calculation.

- Provide a brief mention of the main findings with specific statistical results (e.g., p-values, confidence intervals).

Response: p-values were reported previously. However, confidence intervals have also been included now especially for the reporting of linear regression results in the main body of manuscript as well as the abstract.

**Introduction**

- The introduction effectively establishes the importance of studying the overlap between IBS and SSD and the potential role of stress. However, it could benefit from a more detailed background.

Response: A full new introductory paragraph has now been added and addition of few sentences in the previous content to elaborate and logistically explain the relevance of current study by building on previous research.

- **Recommendations:**
  - Expand on the physiological and psychological mechanisms that may link stress and somatization in IBS patients. Include references to relevant theories and previous research.

Response- Physiological and psychological mechanisms that link stress and somatization in IBS patients has now been explained in the first paragraph of the manuscript especially by discussing the hypothalamus-pituitary-adrenal axis and the related concepts. Furthermore, this manuscript is one of the three manuscripts that are likely to come out from the entire grant-funded research. The entire grant funded project is based on the Common-Sense Model of Self-Regulation and future aim is to evaluate the effectiveness of ACT based on the CSM in the self-management of somatization in IBS, SSD, and IBS-SSD. However, that discussion will be done one we publish our findings on the third variable that is coping in association the pathophysiology in these samples. That manuscript will be published in another journal.

- Provide a more comprehensive review of existing literature, highlighting key studies that have investigated the relationship between stress, somatization, and IBS.

Response- No previous study has previously discussed stress, somatization and IBS together. However, some studies discussed these concepts separately. Those studies have now been cited in the manuscript.

**Methods**

- **Sampling Method:**
  - The study mentions the use of purposeful sampling but does not provide detailed criteria for participant selection.
  - **Recommendations:**
    - Clearly describe the inclusion and exclusion criteria for selecting participants. This should include diagnostic criteria for IBS and SSD, as well as any other relevant factors.
- Response- This was previously also explained. However, now we have explained why we chose purposeful. Patients already had the diagnosis from trained physicians either from gastroenterology clinic or psychiatry clinic. Rome IV criteria was used for IBS diagnosis and DSM V criteria was used for SSD diagnosis. This information was also part of the original manuscript under materials and methods.
  - Justify the sample size of 100 participants. A power analysis should be conducted to determine if the sample size is adequate to detect significant effects.

Response- Power analysis was also done before and reported in the original manuscript. However, the way findings were reported created a confusion. Therefore, information has been re-worded. IBS patients, SSD patients, and IBS-SSD patients have been considered separately for analysis. Therefore, G-Power was used to estimate sample sizes needed for generalizability based on statistical tests used. IBS sample in our study was the largest its findings are generalizable to larger populations. It included 67 participants and maximum 55 participants were needed for running ANOVA and 21 participants were only needed for linear regressions. Our sample is larger than both the numbers needed for generalizability.

- **Data Collection Tools:**
  - The use of PHQ-15 and SSRS is appropriate, but their reliability and validity in this specific population are not discussed.
  - **Recommendations:**
    - Discuss the reliability (e.g., Cronbach's alpha) and validity (e.g., construct validity) of the PHQ-15 and SSRS within the context of this study population.

Response- I have calculated the Cronbach alpha for both the tools in the IBS sample now and the reliability is greater than 0.9 for both PHQ-15 and SSRS. I have also updated the manuscript. For the validity, considering the construct validity, both the tools are appropriate as they exactly measure the constructs that were needed in the study. This is one of the reasons that we did not use other tools available and accessible. A small part on this content has been included in the discussion section.

- Mention if these tools have been validated in previous research involving IBS or SSD patients.

Response- Yes, the tools were validated in previous research. This is the content in the original manuscript that explains the validity of SSRS (reference number 23) in the patients with somatization symptoms and the validity of PHQ-15 has been explained by citing reference number 22.

“Convergent validity of the SSRS was calculated by correlating the SSRC scale scores with the somatization sub-scale scores and other sub-scale scores in the Korean version of the SCL-90. and Discriminant validity of the SSRS was calculated by comparing the sub-scales of the healthy study participants with the patient study participants.” PHQ-15 is a shortened version of PHQ that is excessively being used in clinical settings.

- **Statistical Analysis:**
  - The paper mentions the use of SPSS for data analysis but lacks specifics on the statistical tests performed.
  - **Recommendations:**
    - Provide a detailed description of the statistical methods used. This should include descriptive statistics, inferential statistics (e.g., t-tests, ANOVA), and the specific type of linear regression analysis conducted.

Response-Descriptive statistics includes frequencies, and percentages of demographic variables, and means of SSRS scores and PHQ-15 scores. Omnibus, one-way ANOVA was conducted to assess the differences in PHQ scores by education and linear bivariate regression was conducted to assess changes in SSRS scores with the change in PHQ-15 scores.

- Include information on how missing data were handled and any assumptions checked for the regression analysis.

There was no missing data, and assumptions like test of normality, Durbin-Watson statistics, and Collinearity diagnostics were assessed.

**Results**

- **Demographic Information:**
  - The results section does not provide detailed demographic information, which is crucial for understanding the sample.

Response- This section has now been re-written.

- **Recommendations:**
  - Include a table summarizing the demographic characteristics of the sample, such as mean age, gender distribution, and other relevant variables (e.g., duration of illness, comorbid conditions).

Response-	Done.

- **Presentation of Results:**
  - The results for PHQ and SSRS scores are presented but lack comprehensive statistical details.
  - **Recommendations:**
    - Present the range, mean, and standard deviation of PHQ and SSRS scores for each group (IBS, SSD, IBS with SSD).

Response- Done

- Include regression coefficients, confidence intervals, and p-values for the linear regression analysis. This will provide a clearer understanding of the relationship between stress and somatization symptoms.

Response- Done

- **Interpretation of Findings:**
  - The results indicate a stronger correlation between stress-related somatic symptoms and somatization in elderly patients but lack a detailed discussion on this point.
  - **Recommendations:**
    - Discuss possible reasons why stress-related somatic symptoms are more pronounced in elderly IBS patients. This could include factors such as age-related physiological changes, comorbidities, or social factors.
- Response- This was included in the original manuscript also. It is in the discussion section. It is the second-last paragraph under discussion.
  - Consider exploring whether the severity of IBS symptoms moderates the relationship between stress and somatization.

Response- This point has now been discussed in the first paragraph by citing a reference to a study by Benette. However, a complete discussion on this point can be done after this study as the original plan was to first do a survey on the needs assessment of our priority population. Then after the completion of this study, assess if stress can be used as a moderator by assessing through the Path analysis model. It would be a good idea to consider the severity of IBS symptoms as a moderator variable instead. However, this is beyond the scope of the current study. Irrespectively, severity of ibs symptoms is important and has been discussed in relation to stress and somatization in IBS sample.

**Conclusion**

- The conclusion summarizes the main findings and suggests targeted psycho-social interventions but lacks a discussion on limitations and future research directions.

Response- This section has now been included.

- **Recommendations:**
  - Discuss the limitations of the study. These could include the sample size, the use of self-reported measures, and the generalizability of the findings.

Response- Done

- Suggest directions for future research. This could involve longitudinal studies to assess causality, exploring interventions in a more diverse population, or examining the impact of different types of stress on somatization symptoms in IBS patients.

Response- Done.

**Reviewer 2**

• **Stratify Participant Groups**:

- Consider stratifying the sample into more specific subgroups (e.g., IBS only, SSD only, IBS with SSD) to reduce heterogeneity and allow for clearer insights into each group’s symptomatology and stress responses.

Response- Acknowledged. Sorry about the confusion created through the original manuscript. Analysis have been done separately. These were three different samples from three different populations. Only IBS sample was large enough for generalizability. However, as the analysis were done on all the samples and were significant, it was appropriate to report the findings of all the samples. The wording “sub-groups” was inaccurate and now has been replaced with “sample” or samples as needed.

• **Detail Statistical Methods**:

- Provide a comprehensive description of all statistical methods used, including the rationale for their selection, to enhance transparency and reproducibility.

Response- Done in the manuscript now.

• **Enhance Results Presentation**:

- Report additional statistical details such as confidence intervals, p-values, regression coefficients, and R-squared values to provide a more complete understanding of the findings.

Response- Done now.

**Contextualize Findings with Literature**:

- Include a robust literature review to situate the study’s findings within the broader context of existing research, enhancing the discussion and highlighting the study’s contribution.

Response- Additional information has now been included.
